# Retraction: Comparative Effectiveness of Health Communication Strategies in Nursing: A Mixed Methods Study of Internet, mHealth, and Social Media Versus Traditional Methods

**DOI:** 10.2196/89640

**Published:** 2026-02-04

**Authors:** JMIR Publications Editorial Office

**Affiliations:** 1 JMIR Publications, 130 Queens Quay E, Suite 1100, Toronto, ON, M5A 0P6, Canada, 1 416 583 2040

The JMIR Publications Editorial Office is retracting the article “Comparative Effectiveness of Health Communication Strategies in Nursing: A Mixed Methods Study of Internet, mHealth, and Social Media Versus Traditional Methods” by Hamarash et al [1]. This follows an investigation that identified concerns regarding the integrity of the peer-review process that occurred for this article. We regret that these issues were not identified prior to publication.

All authors did not agree with retraction.
